# Who Is Using Outdoor Fitness Equipment and How? The Case of Xihu Park

**DOI:** 10.3390/ijerph14040448

**Published:** 2017-04-21

**Authors:** Hsueh-wen Chow, Andrew J. Mowen, Guan-lin Wu

**Affiliations:** 1 Graduate Institute of Physical Education, Health and Leisure Studies, National Cheng Kung University, Tainan 701, Taiwan; allen78416@gmail.com; 2 Department of Recreation, Park and Tourism Management, The Pennsylvania State University, University Park, PA 16802, USA; ajm194@psu.edu

**Keywords:** observational study, physical activity, outdoor gym, video monitoring, fitness zones

## Abstract

Outdoor fitness equipment (OFE) placed in public parks has the potential to encourage physical activity. However, little is known about OFE users and use patterns. This study employed onsite and video observations of OFE usage to describe user characteristics and patterns in Xihu Park. Results indicate that OFE in this park attracted considerable use, particularly in the early morning and late afternoon. During these peak-hour observations, approximately 12 users per hour used the OFE, with the majority being females and seniors. The triple arm stretch and air walker were the most popular stations. However, most OFE users interacted with less than three of the available six OFE stations. Furthermore, users spent an average of less than nine minutes on all OFE stations combined. While OFE equipment was well-used in this urban park, it appears users did not interact with OFE at rates to produce a sufficient bout or level of physical activity during their park visit. Further investigations of OFE are encouraged to determine their health impact.

## 1. Introduction

A sedentary lifestyle has a significant negative impact on health due to its linkage with obesity and chronic disease, both of which are associated with high medical costs [1]. Although the health benefits of increased physical activity are well-documented in the literature [2], achieving sufficient levels of physical activity and realizing its benefits across a broad population remains a persistent public health challenge [3]. According to the socio-ecological model [4,5], the built environment is an important contributor to physical activity across broad populations and represents a more permanent approach to increase physical activity than targeted, but short-lived intervention programs. A wide range of studies have investigated the built environment’s influence on shaping the public’s physical activity and health, which generally found positive associations [6,7,8,9,10,11].

One feature of the built environment, public parks, are often situated close to population centers and provide opportunities for a range of activities, including those associated with moderate-to-vigorous physical activity (MVPA) [12,13]. Consequently, parks have been identified as ideal settings to promote physical activity through a range of physical, policy, and programmatic strategies [14,15,16]. Prior literature identified factors of parks that influence visitation and physical activity, including ease of access, distance, locations, sizes, amenities, social environment, trails, organized activities or events, and nature orientation [17,18,19,20,21].

Park visitors have been found to engage in various levels of physical activities. For example, McKenzie et al. [22] observed a total of 16,244 individuals in eight parks in the Los Angeles area and found that most visitors engaged in sedentary activities (such as lying down, sitting on benches, chatting with friends) and these behaviors are especially frequent among females (71%), compared to males (62%). McKenzie et al. further investigated the existence of differences between areas’ types and visitors’ levels of physical activity. The results determined that vigorous physical activity more likely occurred in multi-purpose fields and less likely in picnic areas.

Much of the initial park-based physical activity research focused on general park characteristics (e.g., distance, park size, park type) with respect to physical activity, as opposed to specific types of park features/amenities offered in these spaces. Subsequently, however, researchers have assessed the contribution of specific park features and design considerations on physical activity [23,24]. For example, Besenyi et al. [25] conducted studies in four parks in Kansas City and found that paved trails and tennis courts were active areas for adult visitors to parks, and playgrounds were active venues for children. One such facility/activity feature is the growing installation of outdoor fitness equipment (OFE) within parks and other public spaces [26]. OFE are exercise machines that mimic those found in traditional indoor gyms and rehabilitation clinics. OFE typically provide free access for the general public to exercise in outdoor settings such as parks, community plazas, or schools. Generally, an OFE zone is composed several mechanical stations with specific fitness purposes or targeting at certain area of a body [27]. For example, the arm stretch station is designed to improve flexibility focusing on the upper limb area.

OFE in public parks has gained international popularity, particularly in Taiwan and other Asian countries [28]. According to an investigation by Chow [29], more than half of the parks in the cities of Taipei and Tainan have installed OFE, and a growing number of OFE is being added to parks in the United States [30], in European countries such as Spain [27] and Portugal [31], in south America [32], and in Australia [33,34].

These examples document the rapid spread of OFE, and this popularity is seemingly a response to demand for more amenities to increase physical activity. As such, OFE has been the target of a handful of studies to evaluate their potential in promoting physical activity. For example, Cohen et al. [35] evaluated 12 parks with OFE to compare the number of users and users’ energy expenditures. They found that parks with OFE attracted more first-time visitors and were associated with higher energy expenditures than parks without OFE. However, the number of total park visitors was not significantly higher at OFE parks than non-OFE parks. Another study observed a higher number of users in the time immediately following OFE installation, but not at a longer, 12-month follow-up period [36].

Globally, OFE is increasing in its popularity with the assumption that they offer the public an avenue to increase their physical activity and may also improve other health outcomes (e.g., flexibility, balance, and strength). However, there is limited evidence that OFE in parks are associated with enhanced physical activity [34,36,37]. Although in Cohen et al. [35] and Cranney et al. [36] associated use of OFE with MVPA, further study regarding OFE users, use, and physical activity behaviors is warranted.

Specifically, it is important to assess OFE physical activity in terms of four principal components of exercise behaviors—frequency, intensity, duration, and type of physical activity (FITT) [38]. To date, little is known about the characteristics of OFE users and their use patterns in terms of FITT. As OFE increasingly spreads to parks over a range of communities and countries, it is important to understand how OFE are used, who is using it, and how much physical activity occurs there. Such evidence could provide information for further or fewer installations. Given that age and gender differences exist in overall and different types of physical activities [39,40,41], it is also important to explore if there are any age or gender differences in the use of OFE.

In Taiwan, there are numerous parks where OFE was installed more than a decade ago. Therefore, the study of the OFE users’ behavior in a Taiwan park could eliminate the bias of novelty [36]. Assessing OFE use and user characteristics in this context could provide more accurate measurements of the physical activity and important information to better tailor OFE design/installation as well as programming in order to maximize their physical activity and public health potential. To address these gaps, this study (1) assesses OFE user and use characteristics (e.g., gender, age, duration of overall OFE use, time of use), (2) identifies the number of OFE users by age group and by time of visit, (3) examines if there are differences in terms of OFE duration of use across different gender, age groups, and across the different stations or types of OFE, and (4) documents the number of users for each individual OFE station and the duration of using each OFE station.

## 2. Materials and Methods

### 2.1. Case Study Park

The case study site was in Xihu Park in Tainan, located in southern Taiwan, a city with a population of 1.8 million. The size of the park is 0.84 acres. The neighborhood population in this district is approximately 40,180, with a high density of aging population (28%) older than 65 years. Since weather greatly influences outdoor activities [19,42], the characteristics of the selected sites during the study are important to note: the mean temperature in Tainan was 28.5 °C (min: 22.9 °C ~max: 34.2 °C), five rainy days occurred in September 2012 (number of days with precipitation ≥0.1 mm), and the mean relative humidity was 68 mm [43].

### 2.2. Data Collection

#### 2.2.1. First Stage: Day-Long Observation Using SOPARC

This study followed several steps to collect data that best capture OFE users’ behaviors. First, determining patterns during peak times of OFE use was assessed through the System for Observing Play and Recreation in Communities (SOPARC) [22] with minor modifications (excluding ethnicity and intensity of physical activity). SOPARC is an established, valid, and reliable observational technique for assessing physical activity in open recreational spaces and parks [22,35,44]. SOPARC observations typically record the number of users and user’s genders, age groupings, ethnicities, as well as physical activity intensity. The current study, however, assessed only gender and age group (age group: child (2–12 years), teenager (13–20 years), adult (21–59 years), or senior (60+ years)) [45], since there is an obvious lack of racial/ethnic diversity in Taiwan, with 95% of Taiwanese belonging to the Han people [46]. A primary purpose of SOPARC is to assess physical activity levels in parks. However, currently no scientific measure of OFE intensity is available. Thus, physical activity intensity was not assessed in this study.

To achieve greater SOPARC data consensus among observers, a two-hour laboratory training session and a three-hour on-site session familiarized assistants with the SOPARC manual and field techniques. An independent observer simultaneously recorded data and agreement among all subjects, established the following reliability: 100% for gender, 92% for age group, and 91% for number of users. 

To collect data on the number of persons using the OFE, observers visited the pilot study’s park during two weekday and two weekend sessions for 15 h of continuous observation from 6:00 am to 9:00 pm. After identifying peak use periods, which classified more than ten users, several trials of direct observation captured OFE users’ behavior. The SOPARC protocol involved counting the number of users of the overall OFE zone and documented gender and age groups.

#### 2.2.2. Second Stage: Peak Hour Observation Using Video Camera

Observers reported difficultly assessing the number of users due to crowded conditions during the peak periods, which biases observational results. The SOPARC protocol also identifies this limitation [22]. To overcome the limitation and gain further detailed information of users’ of OFE as well as the duration of using each of the OFE station, video observation supplemented direct observations. With technological advancements, video monitoring has emerged as a use monitoring tool to capture visitation patterns at recreational sites [47]. Video monitoring has several benefits, such as identifying groups’ sizes and temporal patterns, and tracking explicit users’ behaviors—key pieces of information for proactive management and understanding park use [48,49].

During the onsite observations in this study, the research team (at least two observers) employed two digital video cameras simultaneously from two angles of the OFE area to capture the entire OFE zone, allowing observation of actual use of specific OFE, for how long, and by whom. The advantages of using video observations include avoiding bias among observers, capturing exact durations of operating each equipment, and identifying the users’ patterns. The data consisted of on-site logs and video data, analyzed, off-site. Observers identified each OFE user’s gender, age group, and recorded in logs the specific equipment used. A sign reading “video observation: study in progress” placed on site notified the park’s visitors of the research in progress. The trained observers willingly responded to any questions visitors raised. A limited access computer stored the videos. The National Cheng Kung University Internal Review Board (IRB_ER-99-375) provided ethical approval for this study and its protocol. As guided from the human research protections policy of Ministry of Science and Technology Regulations in Taiwan [50] and U.S. Department of Health & Human Service [51], observed behaviors in public places represent data from public domain. As such, no informed consent from subjects was necessary and the study qualifies for exemption.

Video recording sessions were conducted nine weekdays and two weekends in September 2012. Each day of observation was during peak periods mornings (6:30–8:30) and afternoons (15:30–17:30), except one afternoon (15 September 2012) due to rain. Each session lasted two hours, resulting in 42 h of video observations for analysis. 

### 2.3. Analysis

Inter-rater reliability assessed agreement between independent raters for both onsite observation and for video data analysis using aggregation of overall agreement proportion and Pearson correlation coefficient [22].

Descriptive statistics were used to assess the profile of OFE users in terms of gender and age from day-long observations during peak times of use. Then, synchronizing video data from the two video-cameras and comparison with onsite observations’ logs identified the subjects in the videos. Next, linking each subject on a video identified use of each OFE and the duration of use, leading to calculation of overall use and duration. Two trained assistants individually analyzed the video data from two sessions (one morning and one afternoon) followed by inter-rater reliability assessment. Having established a high level of agreement for inter-rater reliability (IRR: Cohen Kappa = 0.96), only one coder analyzed other sessions, individually, due to the requirement of five to eight hours to analyze a one-hour video (dependent on the number of OFE users in a session) as reviewing required several repetitions and pauses to record exact times users began or ceased using a piece of equipment. Finally, the total time of use in terms of different gender, age group, and observation time of the day were compared using one-way ANOVA or Krurskal-Wallis tests for nonparametric data. The correlation of number of OFE used and total time of use was conducted with Person correlation. The level of significance was set at *p* < 0.05.

## 3. Results

### 3.1. Day-Long Observations

**Figure 1:**
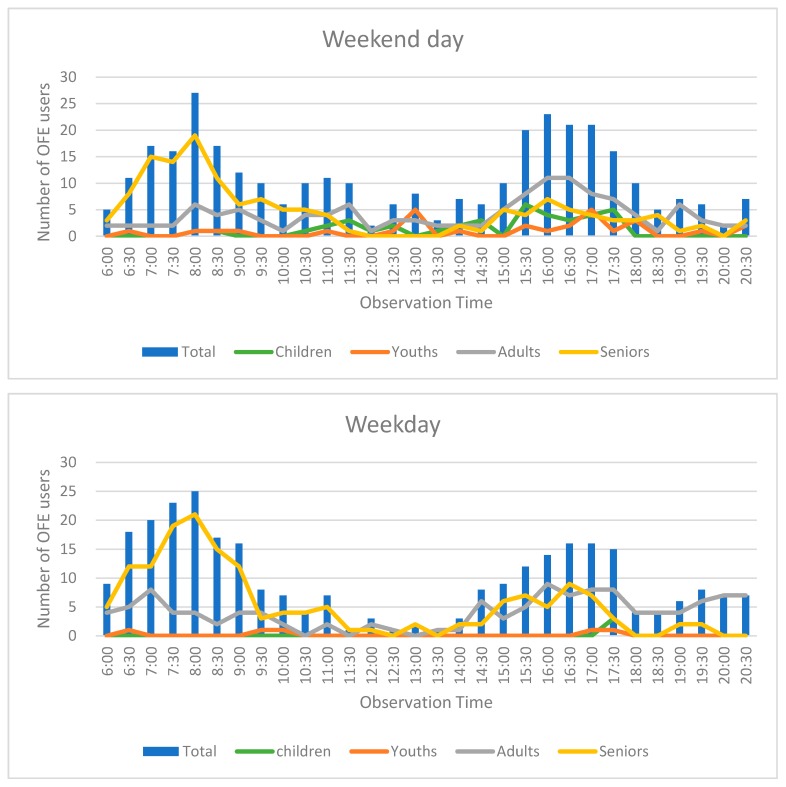
Average number of users of outdoor fitness equipment (OFE) by time of day—weekend vs. weekday.

Two periods, early morning from 6:30 to 8:30 and during sunset between 15:30 to 17:30, attracted the most OFE users (see Figure 1). The pattern was similar on weekdays and weekends. With respect to age groups, seniors accounted almost half (49%) of total OFE users, followed by adults (39%), while youths were the fewest (5%). Throughout the day, different age groups displayed varying patterns: seniors clustered in the morning (before 9:00), but the other groups tended to use the OFE in late afternoons. Very few persons appeared during midday (11:30–14:00). Cross-tabular analysis (chi-square) using R software (The R Foundation, version 3.0.2, Vienna, Austria) for the Fisher exact test (since one cell is less than 5) established a significant difference between OFE visitors during weekdays/weekends and among different age groups (see Table 1). More OFE users appeared on weekends than weekdays, with children and youths being more prevalent than seniors.

### 3.2. OFE Use Patterns

The 42 h of video observations identified 500 individuals as OFE users in Xihu Park. Six individuals were excluded from analysis due to undetermined age group or gender. Observation identified a mean of 24 users (range: 13–45) per two-hour observation session, which means an average of 12 users per hour. More users were observed during morning sessions than during afternoons. Of these, females accounted for more than 71% users. With respect to age groups, similar day-long observations found seniors to account for more than half of all users (52.5%), followed by adults (33.4%), children (11.2%), and youths (2%). There were six OFE stations in the park. However, most visitors (79.9%) used fewer than three stations of equipment. Only 3.6% used all six stations of equipment during observations (see Table 2). As the data of total time of use violated the assumption of normality in commonly-used parametric tests (e.g., ANOVA), non-parametric Kruskal-Wallis tests were used to analyze differences between groups [52]. Both mean and median time of use were reported. Results shows that no difference exists for total time of use, according to gender or time of day. However, the time children use OFE was significantly less than adults or seniors. The more OFE stations an individual used, the longer the time they were observed to remain at the whole area of the OFE (Pearson r = 0.469, *p* < 0.001) (Table 2).

In terms of amount of use for each station of OFE, triple arm stretch attracts the most users. The popularity’ ranking by number of users of the other OFE is: (2) air walker, (3) triple waist twister, (4) single bonny rider, (5) shoulder wheel, and (6) arm wheel. The average duration of each OFE’s use followed the same order as popularity. However, on average, each equipment’s use was less than five minutes. On average, the total time spent using all equipment, per individual was less than nine minutes (Table 3).

## 4. Discussion

This study identified the demographics of OFE users and patterns of use Xihu Park in Taiwan. This park attracted a substantial number of OFE users. The majority use the equipment in the early mornings and afternoons. More users were observed in the weekend than during the weekday which was similar to prior OFE studies [35]. During peak hours of observation, approximately 12 users were present per hour. Most users were females and seniors, and most users interacted with fewer than three of the six OFE stations present in this park. The two most popular OFEs were the Triple Arm Stretch and Air Walker. This is consistent with earlier qualitative research, which indicate many seniors use OFE for rehabilitation purposes [37]. In contrast, Cohen et al. [35] reported most the frequently used OFEs were dual pendulum, the ski machine, and the leg press.

In the study by Cranney [36], data from observations and interviews were used. In their observation data, the most frequently used OFE were (1) pull down, (2) elliptical trainer, (3) aerobic cycle, and (4) parallel bars. From the interview responses, the most frequently used OFE were (1) pull down, (2) chest press, and (3) elliptical trainer. These findings, however, are difficult to compare with the current study, given the differences in available OFE across the studies. Another reason might be attributable to the preferences of different types of exercise favored by visitors from various backgrounds such as ethnicity, culture or environmental issues [42,53].

On average, each OFE user operated one device for less than five minutes with a total time for using all equipment less than nine minutes. No difference existed for total time of use based on gender, or time of observation. However, the time children use OFE was significantly less than the time spent by adults and seniors. These results contradict the findings of Mora [32] who found most respondents reported spending more than 15 min using OFE. The tendency of overestimating of self-reported physical activity has been reported in several studies and over-estimating OFE use may also be subject to the same criticism [40,54]. The strength of the present study is in the use of video-recorded data to more accurately quantify the durations and patterns of OFE users in a park context (natural experiment). This method helps to avoid subjective recall bias from participants and render more objective, accurate measures of OFE use behaviors.

From this study’s data, it appears as that, while OFE is a popular feature within this park (and one that may serve to encourage visitation), visitors may not be using the equipment long enough to achieve physical activity benefits from using OFE in these parks. Perhaps visitors could achieve the recommended level of physical activity from other park activities such as jogging, walking, ball games, or other programmed activities while traveling to or visiting the park. Nevertheless, as stated by Chow [37], OFE seems to be perceived as an “additional feature that is fun to use” rather than a resource for “exercise.” There is currently no standard exercise prescription for use of OFE, although some manufacturers have provided guidelines. For example, Landscape Structures Inc. [55] suggested three levels (beginning, intermediate, advanced) or routines of using OFE with five minutes of cardio OFE warm-up, several sets of 8–15 repetition resistant training with 30 s rest in between, and a five-minute cool down session. Although the total suggested duration for this routine is not available, these and other OFE use recommendations should consider incorporating at least a ten minute bout as recommended by the American College of Sports and Medicine for a shorter session [56].

The results indicate seniors account for a major proportion of OFE users in this park-consistent with study by Cranney et al. [36]. These results are different from those of a study by Cohen et al. [35] and Bettencourt and Neves [31], who found adults and children to be the majority users, respectively. One explanation for this discrepancy could be differences in ethnicity, culture, or environmental issues [42,53]. For example, Cranney et al. [36] observed that a high proportion of OFE users spoke a language other than English. Another possible reason for lack of seniors seen using OFE in other studies might be a reflection of seniors being a minority in visiting the park in general. For example, Cohen investigated 174 neighborhood parks in the U.S. and found that seniors represented only 4% of park users.

A national survey conducted in Taiwan revealed that about 85% of Taiwanese report prefer outdoor physical activities than indoors. Additionally, 82% of population report no need to pay for participating in exercise/physical activity [57]. OFE responds to the demand for more affordable exercise facilities for seniors [58]. In addition, since this study identified females’ reliance on local physical activity facilities compared to males [59], methods for attracting older male users of OFE facilities are worthy consideration for future marketing/outreach efforts.

Regarding OFE users’ behaviors, it was found that OFE users did not use all equipment while visiting OFE at the park. The most popular station was the arm stretch machine, which was also identified in a prior qualitative study by many seniors to increase their flexibility and ease common shoulder problems [37]. The duration of individual OFE use was rather brief. For example, on average, use of the air-walker was only four minutes per individual, and the average time for total OFE use was less than nine minutes. These durations were insufficient since most aerobic exercises recommend a minimum of 30–60 min of moderate-intensity or 20–60 min of vigorous-intensity to be effective [56]. An earlier study [29] inspected all OFE facilities in Taipei and Tainan parks and found inadequate instructional labels for proper use of equipment and recommended duration/repetition. However, most OFE users observed others’ activities and developed individualized methods for themselves. The reason for insufficient duration may arise from a lack of knowledge, becoming bored, or due to crowding/congestion in OFE zones. Researchers have suggested that more personal instructions or on-site labeling may assist increasing efficient use of OFE to achieve positive health outcomes [36,37]. In addition, providing a wide range of OFE equipment/stations, which include different training functions such as balance, strength, mobility, and coordination, may attract users to take advantage of more than four OFE stations during their visits, thereby leading to 10 or more minutes of use, as documented in this study. Despite these apparent deficiencies, it is important to acknowledge that OFE may be a park attraction that motivates higher park activity levels than parks without such features and might even encourage people to walk (or be active) to travel to these parks.

Recently, sports and fitness equipment companies (i.e., Landscape Structure Inc.’s HealthBeat^®^ User Guide) [55] and health promotion agencies (i.e., the Health Promotion Service’s How to Use an Outdoor Gym) [60] have begun to issue guidelines for proper use of these OFE, and these recommendations deserve further investigation in terms of their efficacy.

Another reason for short OFE duration could be that using the equipment might not be the primary park activity. As other studies indicate, OFE users could also engage in walking, cycling or group exercising while visiting parks [32,37]. Therefore, using OFE is not the main physical activity resources for park visitors, but often serves as an add-on feature—to increase park-based physical activity levels.

## 5. Conclusions

Better understanding OFE users’ demographics and patterns are important considerations to improve the impact of public parks on physical activity and other health outcomes. This case study contributes to the limited but growing evidence on OFE users’ behaviors, and used objective measures (video observation techniques) to document use characteristics of OFE in Taiwan. This study found that OFE users did not use OFE long enough during their time interacting with OFE to meet the minimum bout duration for physical activity recommendations. Thus, the benefits of OFE alone in achieving a higher level of physical activity to improve health remains questionable. However, given the popularity of specific OFE among seniors, other important health benefits could be realized by having OFE in parks (e.g., improved flexibility and muscle tone, and increased social interactions among seniors).

This study had several limitations. First, since this pilot study only selects one park as a case, generalization for other parks in other cities or for parks of different sizes and scopes may not be appropriate. Since seasonal variations exist for park visitors, the current study, which collected data only during one season, suggests expansion of research for other seasons. The basis for judging subjects’ age groups was by appearance and may introduce bias in the results. Video observations occurred only during peak periods of activity to maximize documenting OFE users’ patterns. Future studies could expand hours of observation to full-days to better capture the complete picture of OFE use, if resources are available. Although this study adopted video monitoring to overcome limitations to accumulating precise data, analysis of post-observation video data is labor intensive; therefore, future studies should employ techniques to assist efficiently analyzing observational data. In addition, analytic protocols require expansion of the details of users’ behaviors in terms of frequency of movements or the intensity of using each station of OFE. Experimental designs for effectiveness of using OFE for beneficial outcomes affecting health need to separate experimental groups (OFE users) from control groups (OFE non-users) to assist understanding the effectiveness of OFE use. The exact intensity of using each OFE in terms of energy expenditure remains undetermined and requires future research. Another interesting aspect would be comparing the differences of duration of OFE use while users are in pairs, with groups, or alone. Likewise, it would be interesting to examine or compare intensity of exercise (e.g., heart rate) while using OFE.

Despite these limitations, information derived from this study provides important data for developing strategies to promote OFE for physical activity among park users, especially for seniors. For example, park authorities could host sessions to provide users with instructions for using OFE or could include programmed activities that involve at least 10 min of equipment use. Given the growing popularity of OFE, more information regarding users and their behavioral patterns will help to inform park-based OFE design, programming and management.

## Figures and Tables

**Table 1:** Number of OFE users observed in Xihu Park, Tainan, Taiwan, according to age groups during weekdays and weekends from day-long observations.

Age Group	Weekend	Weekday	Sub-Total
Children	39	3	42
Youths	29	5	34
Adults	122	122	244
Seniors	142	161	303
Total	332	291	623

Fisher exact test (since a cell number is less than 5), *p* < 0.05.

**Table 2:** OFE users’ characteristics and patterns of use and comparison of total time used.

Variable	Number	%	Total Time of Use ^1^		*p*	Post-Hoc
			Mean (S.D.)	Median		
Gender						
Males	140	28.3	8:02 (7:10)	6:20	0.053 ^2^	
Females	355	71.7	9:20 (8:06)	7:07		
Age group						
Children	56	11.3	5:15 (5:07)	3:03	<0.001 ^2^	children < adults
Youth	10	2	7:43 (6:56)	6:00		children < seniors
Adults	169	34.1	8:57 (8:25)	6:00		
Seniors	260	52.5	9:49 (7:48)	7:42		
Time observed						
Morning	294	58.8	8:52 (6:55)	6:52	0.38 ^2^	
Afternoon	205	41.2	9:02 (9:02)	6:07		
Number of OFE used						
0	2	0.4	0 (0)	0	<0.001 ^3^	*r* = 0.469
1	155	31.1	5:01 (7:19)	2:28		
2	129	25.9	8:30 (7:38)	6:11		
3	114	22.9	9:45 (7:03)	7:41		
4	51	10.2	12:02 (6:02)	11:03		
5	30	6.0	16:57 (7:03)	15:53		
6	18	3.6	19:21 (9:18)	18:13		

^1^ Time of use stands for minutes: seconds; ^2^ *p*-values for comparison between groups by non-parametric analysis of Kruskal Wallis; ^3^ *p*-value for Pearson Correlation Coefficient.

**Table 3:** Number of users and operation duration for individual OFE in Xihu Park.

Name of Equipment	Number of Users	Average Number of Users per Piece	Average Operation Duration (mm:ss)	Max. Operation Duration (mm:ss)	Min. Operation Duration (mm:ss)
Triple arm stretch	297	99	4:40	28:00	0:05
Air walker	262	131	4:05	18:30	0:10
Triple waist Twister	249	83	3:48	26:12	0:05
Single bonny rider	148	148	3:17	24:27	0:05
Shoulder wheel	138	69	2:21	9:10	0:06
Arm wheel	122	61	2:01	9:21	0:05
Total			8:55	54:13	

(mm:ss) stands for minutes: seconds.
